# Functionalized Periodic Mesoporous Organosilicas: Tunable Hydrophobic Solid Acids for Biomass Conversion

**DOI:** 10.3390/molecules24020239

**Published:** 2019-01-10

**Authors:** Jinesh C. Manayil, Adam F. Lee, Karen Wilson

**Affiliations:** 1 European Bioenergy Research Institute (EBRI), Aston University, Birmingham B4 7ET, UK; j.manayil@aston.ac.uk; 2 Applied Chemistry & Environmental Science, School of Science, RMIT University, 124 La Trobe Street, Melbourne VIC 3001, Australia; adam.lee2@rmit.edu.au

**Keywords:** solid acid catalysis, hydrophobicity, periodic mesoporous organosilica, biorefining, biofuels

## Abstract

The catalytic deoxygenation of bio-based feedstocks to fuels and chemicals presents new challenges to the catalytic scientist, with many transformations either performed in or liberating water as a byproduct during reaction. The design of catalysts with tunable hydrophobicity to aid product and reactant adsorption or desorption, respectively, is vital for processes including (trans)esterification and condensation reactions employed in sustainable biodiesel production and bio-oil upgrading processes. Increasing surface hydrophobicity of catalyst materials offers a means to displace water from the catalyst active site, and minimizes potential deactivation or hydrolysis side reactions. Hybrid organic–inorganic porous solids offer exciting opportunities to tune surface polarity and hydrophobicity, as well as critical parameters in controlling adsorption, reactant activation, and product selectivity in liquid and vapor phase catalysis. Here, we review advances in the synthesis and application of sulfonic-acid-functionalized periodic mesoporous organosilicas (PMO) as tunable hydrophobic solid acid catalysts in reactions relevant to biorefining and biofuel production.

## 1. Introduction

Tackling global warming associated with anthropogenic carbon emissions from fossil fuel utilization is a grand challenge for both developed and developing nations in the 21st century. Bioderived feedstocks obtained from waste or non-food-derived lignocellulose, sugar, or oleaginous resources are potential low-cost, carbon-neutral replacements for fossil-oil-derived transportation fuels and organic chemicals [1,2]. Coproduction of biofuels and chemicals through a biorefinery approach in which high-volume/low-value (fuels and commodity chemicals) products are produced in tandem with low-volume/high-value (fine/specialty chemicals) products [3] offers the most economically viable route to valorize biomass. Catalysis played a critical role in the development of the petrochemical industry, and in processes to manufacture chemical intermediates, industrial products, and materials ubiquitous in modern society [4,5], and will likewise play a fundamental role in underpinning future biorefinery technology.

**Scheme 1:**
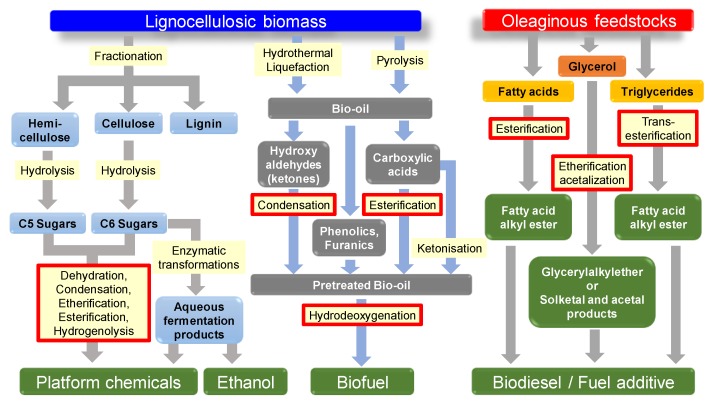
Conversion of bioderived feedstocks to fuels and chemicals, illustrating routes (in red-outlined boxes) where hydrophobic catalysts are of importance in aqueous-phase processing of sugars, bio-oil pretreatment and upgrading, and utilization of oleaginous feedstocks. While transesterification of triglycerides does not produce water, high water contents in waste oil can lead to undesired triglyceride or ester hydrolysis.

Bio-based feedstocks are highly oxygenated, and their conversion processes are most effectively performed at low temperature (<200 °C) in the aqueous phase or in polar solvents [6]; these conditions are very different to hydrocarbon processing in petroleum refineries, where vapor phase processes >400 °C are common. Thus, the utilization of biomass-derived chemicals, whether from lignocellulose, sugar, and oleaginous feedstocks, or fermentation broths, represents an area with extensive research and development (R&D) potential for a renewable feedstock-based technology platform (Scheme 1). Low-temperature (<200 °C) aqueous-phase processing of sugars is an attractive route to produce functional intermediates including aldehydes, alcohols, acids, and esters, via selective deoxygenation and functionalization of (hemi)cellulosic-derived sugars derived from chemical or enzymatic hydrolysis. While fast pyrolysis and hydrothermal liquefaction of waste lignocellulose are widely employed to produce bio-oils, resulting oils are comprised of carboxylic acids, alcohols, furans, aldehydes, esters, ketones, phenolics, and sugars, and retain a high oxygen content. Pyrolysis oils are also highly acidic and corrosive (pH 2–3), with reactive oxygenates including hydroxyacetic acid, hydroxyacetaldehyde, and hydroxyacetone, unstable toward polymerization. Ketonization, aldol condensation, and esterification are important pretreatment steps to increase the carbon chain length of small volatile components and reduce the acidity of bio-oils. However, the high water content of bio-oils, and reversible nature of condensation and esterification reactions means hydrophobic catalysts are desirable for these processes. Likewise, conversion of oleaginous feedstocks via (trans)esterification of fatty acids and triglycerides and valorized glycerol byproducts will also necessitate water-tolerant catalysts to facilitate use of cheaper, often high-water-containing, waste oils.

Many classes of solid acid catalyst, spanning hierarchical zeolites, templated mesoporous sulfonic acid silicas, sulfonated carbons, mixed metal oxides, and sulfated, tungstated, and phosphated oxides, were explored for biomass transformation [7,8], the development of water-tolerant and/or hydrophobic porous materials, thus, represents a key challenge for the design catalysts for biorefining [9,10,11]. Generally, hydrophobization of metal-oxide catalysts is achieved via methods to decrease hydroxyl densities during crystallization [11], or surface derivatization with hydrocarbon moieties, as reported for zeolite silylation [12], or ion exchange of hydrotalcites with sodium dodecylsulfate [13], where improved water tolerance is reported. However, control over porosity is limited with such materials, with careful tailoring of pore structure required for transformations of bulky molecules typical of bioderived substrates to minimize mass transport limitations. Mesoporous catalysts with improved hydrothermal stability under aqueous operation are, thus, required offering tunable hydrophobicity to aid reactant adsorption, and eliminate water from the active site [14] during liquid-phase reactions including hydrolysis, dehydration, isomerization, oxidation, (trans)esterification, aldol condensation, hydrogenolysis, and hydrodeoxygenation [15].

Templated mesoporous silica materials are attractive supports to develop heterogeneous catalysts for clean technology applications [16,17,18], with their versatility enabling framework compositions and pore architectures to be readily tuned [19]. A wide range of functionalities can be introduced, spanning metal nanoparticles, framework cations, or organic groups which can be introduced by grafting of organosilanes [20,21,22,23,24,25]. The synthesis of organic–inorganic hybrid materials, enables further introduction of functionality via direct incorporation of organic groups in the walls or on the surface of the templated silica [26,27,28,29,30,31]. However, the availability of tethering sites limits the loading of functional groups achievable via simple grafting routes [32], while attempts to directly incorporate high loadings of organic groups in “one-pot” approaches can lead to loss of structural order during silica templating [33]. Thus, periodic mesoporous organosilicas (PMOs), in which organo-bridged siloxanes are employed to incorporate organic groups within the mesostructured walls [34,35,36], offer an attractive route to introduce hydrophobic characteristics while retaining excellent structural order, and are among the most widely studied organic–inorganic hybrid mesoporous materials, typically possessing high specific surface areas >1000 m^2^/g and pore diameters 1.5–10 nm [37,38,39]. Here, we discuss recent advances in the synthesis of PMO materials, and routes to their derivatization with sulfonic acid centers, and provide an overview of their application in acid-catalyzed reactions and transformations relevant to biorefining and biofuel production. Advanced methods to quantify changes in hydrophobicity are also discussed.

## 2. Periodic Mesoporous Organosilica (PMO) Materials

### 2.1. Synthesis

**Figure 1:**
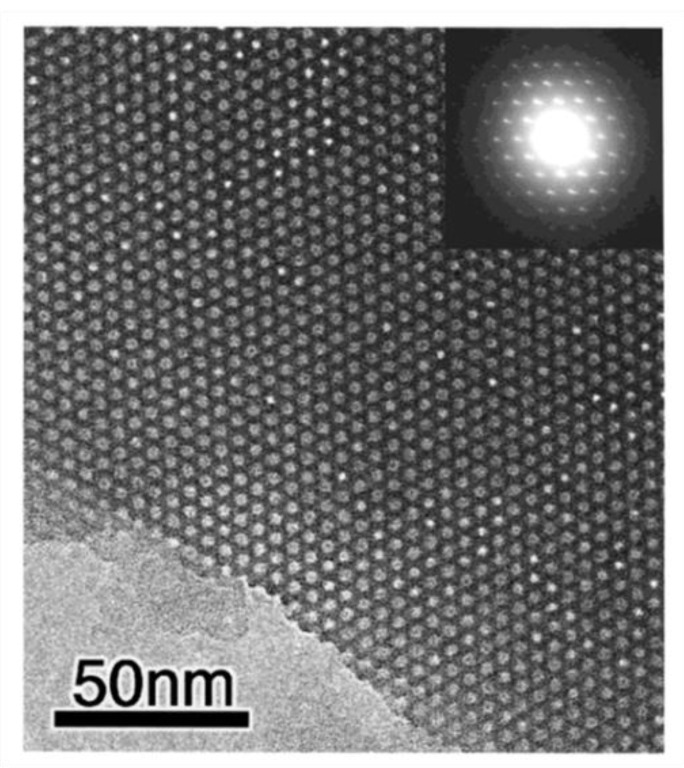
Transmission electron micrograph and electron diffraction pattern (inset) of periodic mesoporous organosilica (PMO) with two-dimensional (2D) hexagonal symmetry. Reprinted with permission from Reference [41]; copyright (1999) American Chemical Society.

**Scheme 2:**
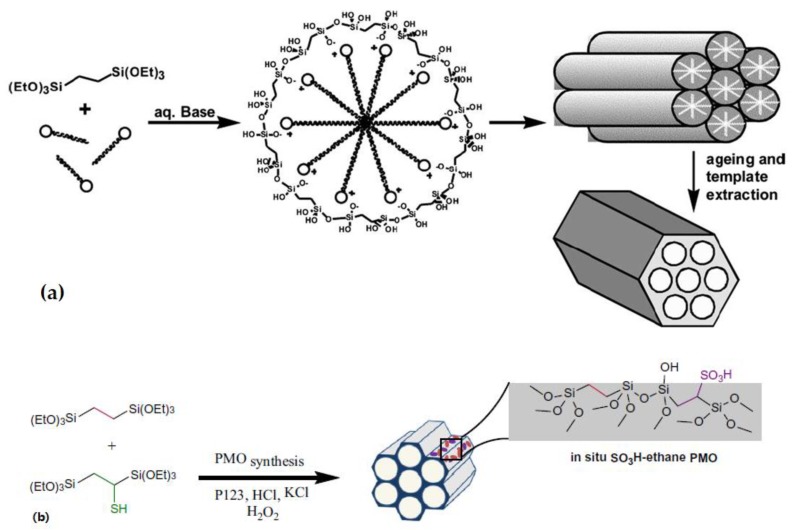
(**a**) Synthesis route of PMOs. Republished with permission from the Royal Society of Chemistry from Reference [51]; permission conveyed through Copyright Clearance Center, Inc. (**b**) Schematic representation of derivatized PMO framework. Reprinted from Reference [68] with permission from Elsevier.

The synthesis of PMOs was first reported in 1999 [40,41,42]; they were explored for their potential applications in catalysis [43], water treatment [44,45], enzyme immobilization [37], gas separation [46], drug delivery [47], and lyophobic systems for intrusion–extrusion of electrolyte solutions [48], as well as in electronics, sensing, biomedical, and chromatography fields [49,50,51,52,53]. PMO synthesis is similar to conventional mesoporous silica (MCM-41, SBA-15), employing liquid crystal templated sol–gel methods, under thermal [54,55,56] or microwave irradiation [57,58], to generate pore networks with 2D-hexagonal symmetry (Figure 1). Organic groups (R^I^) are introduced into the PMO framework using bridged siloxanes ((RO)_3_Si-R^I^-Si(OR)_3_), which are co-condensed with alkoxysilane groups (Si(OR)_4_) which grow around an amphiphilic structure directing agent, to produce hybrid organic–inorganic frameworks (Scheme 2a). Template removal via acid–ethanol or ethanol extraction is normally employed to yield the final PMO [55,59]; however, oxidation treatments using ammonium perchlorate were also reported [60]. Cross-linking of silica in the walls imparts structural rigidity and stability for PMO materials, while the organic framework group introduces flexibility, hydrophobicity, hydrothermal stability, and functionality to the materials (Scheme 2b) [35,36,61,62,63]. PMO materials functionalized with heteroatom-containing organic groups, chiral centers, ionic liquids, and metal complexes were also reported [34,64,65,66,67].

**Scheme 3:**
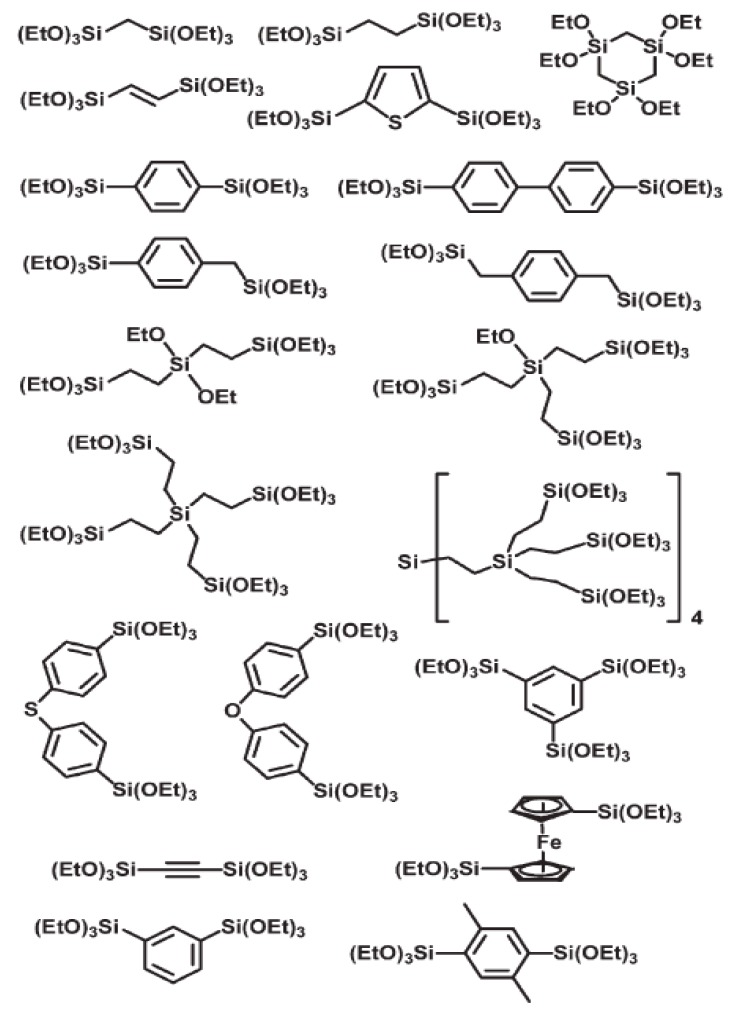
Organic precursors used for PMO synthesis. Republished with permission from the Royal Society of Chemistry from Reference [51]; permission conveyed through Copyright Clearance Center, Inc.

**Scheme 4:**
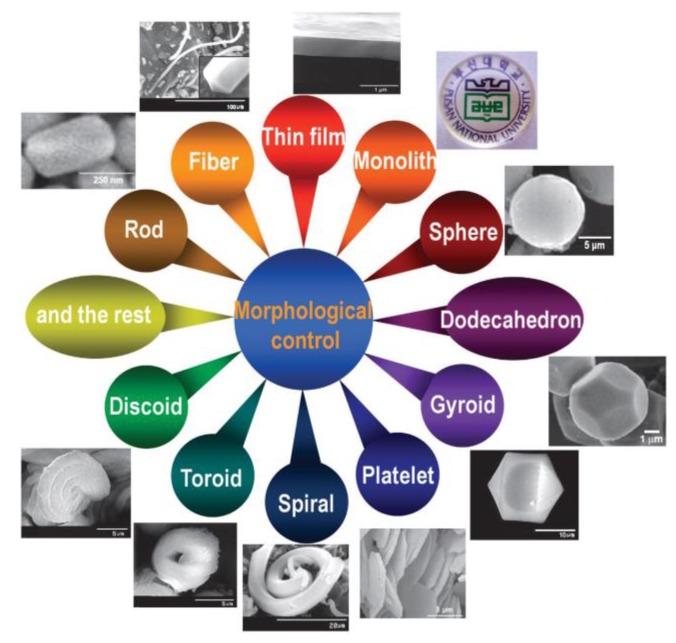
PMO materials with a range of morphologies. Reprinted with permission from Springer Nature Customer Service Centre GmbH: Springer Nature NPG Asia Mater. [34]; copyright (2014).

**Figure 2:**
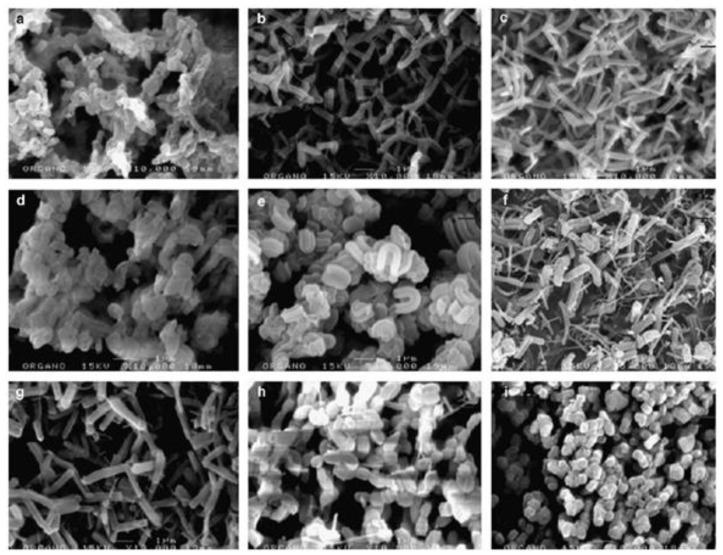
SEM images of PMOs with different morphologies (**a**–**i**). Reprinted from Reference [80]; copyright (2006) with permission from Elsevier.

**Scheme 5:**
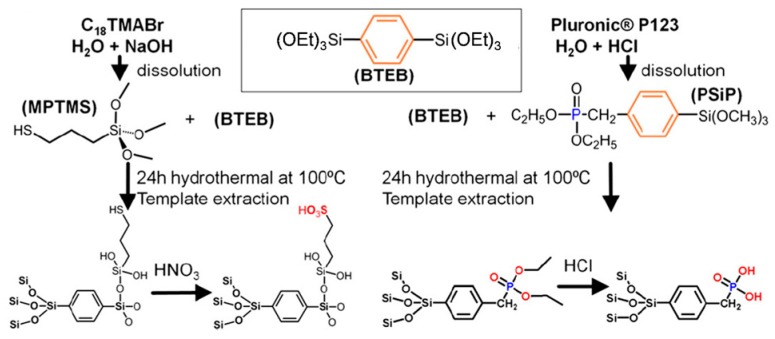
Organic (sulfonic and phosphonic acid) functionalization on PMOs. Reprinted from Reference [52]; copyright (2012) with permission from Elsevier.

Initial reports of PMO materials focused on using simple aliphatic (ethane, ethylene) and aromatic (benzene, biphenyl) bridging groups [27,69]; however, later developments explored the introduction of heteroatom-containing or more complex organic structures such as bridged thiophene [70], thiol [68], heterocyclic tris[3-(trimethoxysilyl)propyl]isocyanurate [71], or chiral benzylic ether-bridged groups [72]. Further functionality can be introduced through co-condensation of bridged siloxanes with amine- [73], mercapto- [73,74], chiral- [65], or cyanopropyl-containing [75] siloxanes into the PMO framework. The successful synthesis of PMO materials is dependent on the structural rigidity of the organic precursor, with Scheme 3 showing a selection of bridged organo-siloxanes used in PMO synthesis [51]. Bi- and trifunctional PMOs were also synthesized using ethyl, phenyl, and thiophene precursors in different molar ratios, with materials with unique rod- and rope-like morphologies produced [76]. The earliest reported PMO materials possessed crystalline frameworks with molecular periodicity of the bridging aromatic siloxanes (e.g., 1,4-*bis-*(triethoxysilyl)benzene, (BTSB)) [77,78,79]. However, this extension of the synthesis to more diverse bridging silanes resulted in loss of long range pore order in the PMO frameworks, which often comprise amorphous pore walls. Fine-tuning of the pore architecture, composition, and morphology can, however, be achieved by varying the ratio of bridging silane (tetramethoxysilane (TMOS) or tetraethoxysilane (TEOS)) in the synthesis media, or via the addition of salt (e.g., KCl) (Scheme 4 and Figure 2) [80,81,82,83,84,85,86,87]. Active sites such as amine, phosphonates, and sulfonic acids (Scheme 5) are readily incorporated via post grafting or co-condensation [73,88,89], for solid base- or acid-catalyzed applications [89,90,91,92,93].

### 2.2. PMO Applications

Tuning the degree of hydrophobicity/hydrophilicity and pore architecture of PMOs proved pivotal for their application in catalysis [43], water treatment [44,45], enzyme immobilization [37], gas separation [46], drug delivery [47], and protein separation/purification [94]. Large-pore phenyl-bridged mesoporous organosilicas were also employed for benzene adsorption, in which the hydrophobicity of phenyl groups inside the pore walls facilitates interaction with benzene and increase adsorption capacity [95].

Organic moieties within the pore architecture of PMOs are more versatile for surface functionalization compared to conventional mesoporous silicas, where derivatization is limited by the availability of reactive silanol groups [86]. Sulfonic-acid-derivatized PMOs find application as chromatographic materials and proton conductors [96,97]. In the latter case, sulfonic acid groups increase the hydrophilicity and proton conductivity of phenyl-bridged PMO. The ion-exchange capacity of sulfonic-acid-derivatized materials also proved beneficial for selective adsorption and recovery of Co^2+^ from artificial sea water [98]. PMO materials with high ion-exchange capacity were synthesized for this application using an *N*,*N*′-diureylenepyridine-*bis*-[(3-propyl)triethoxysilane)] bridging silane, which, when reacted with chlorosulfonic acid, had potential for attachment of four sulfonic acid groups per organic bridge.

Adsorption of enzymes, which possess hydrophobic and hydrophilic domains, and bulky drug molecules, can also be controlled by altering the affinity of the PMO support for water [47]. Lipase and laccase immobilization was explored on two PMO materials with differing hydrophobicity, and electrostatic and hydrogen-bonding interaction capabilities. For lipase, which has a hydrophobic domain, immobilization on a hydrophobic PMO (with C=C bridges) was most successful, while, for laccase, chemical affinity and pore size was important, with an amine-functionalized pore expanded PMO required [37]. PMOs are believed to offer superior performance to conventional mesoporous silicas for enzyme immobilization, owing to the presence of functional groups within the pore walls minimizing steric hindrance within pores or near pore openings, as commonly observed for materials with surface-grafted functional groups [83].

**Scheme 6:**
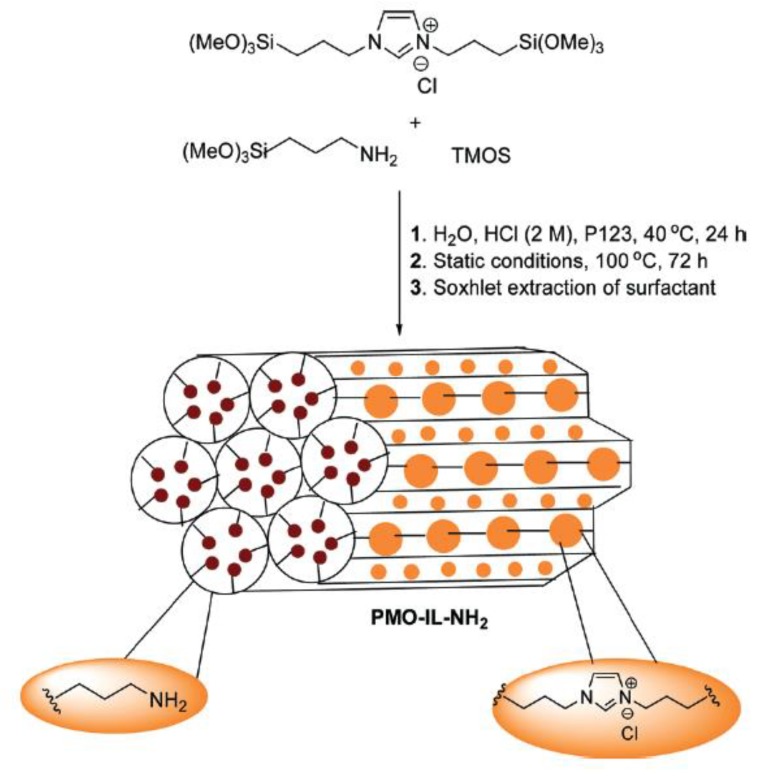
Synthesis of multifunctional PMO materials. Republished with permission from the Royal Society of Chemistry from Reference [93]; permission conveyed through Copyright Clearance Center, Inc.

The synthesis of multifunctional PMO materials (Scheme 6) was also reported to produce bifunctional catalysts in which amine or sulfonic acids are incorporated alongside framework imidazolium groups, which are key components of an ionic liquid [93,99].

The presence of the ionic liquid and amine groups enhances the activity for base-catalyzed Knoevenagel condensation of ethyl cyanoacetate with benzaldehyde. The lipophilicity of ionic liquid is claimed to help diffusion of substrates through the meso channels, while the imidazolium ring activates the aldehydes during reaction [84]. Ionic liquid and sulfonic-acid-containing PMOs were reported for Biginelli condensations [90], and, while the role of the ionic liquid was not articulated, a clear enhancement in reactivity and stability of the bifunctional catalyst was reported.

PMO materials were also employed for metal-complex immobilization, with the activity of Rh and Ru organometallic complexes grafted onto phenyl-bridged PMO materials exhibiting enhanced activity in hydrogenation and sulfoxidation reactions compared to hydrophilic MCM-41 and SBA-15 counterparts. The enhanced activity was attributed to superior diffusion of reactants and desorption of products over PMO material compared to hydrophilic silica materials [66].

## 3. Sulfonic-Acid-Functionalized PMOs

Templated mesoporous sulfonic acid catalysts are attracting significant interest as strong solid Brønsted acidic replacements for corrosive and hazardous mineral acids such as H_2_SO_4_ or HCl in organic synthesis, and offer superior performance to commercial sulfonated polymer resins, including Amberlyst-15, which exhibits poor acid-site accessibility and stability under hydrothermal reaction conditions. Sulfonic acid PMO materials consequently attracted significant interest in Green Chem., and for biomass conversion where solid acids with tunable surface properties are sought; their synthesis and application in acid-catalyzed reactions is reviewed in this section.

### 3.1. Synthesis

Sulfonic-acid-derivatized PMOs are attractive materials exhibiting strong Brønsted acidity for catalytic applications, with sulfonic acid functionalization most commonly achieved via the incorporation and oxidation of organo-thiol or bridged sulfide groups into the PMO framework [68,100,101]. The simplest method to prepare sulfonic-acid-functionalized PMO materials is via one-pot co-condensation [43,74,78,101,102,103] or post-modification grafting [54,59,74,78] with mercaptopropyl trimethoxysilane (MPTMS) followed by oxidation with H_2_O_2_. However, some studies reported incomplete oxidation of thiol groups at higher MPTMS loadings, with the formation of disulfide species observed which can limit accessibility of acid sites; [104] care should, therefore, be taken correlating S content with activity.

**Scheme 7:**
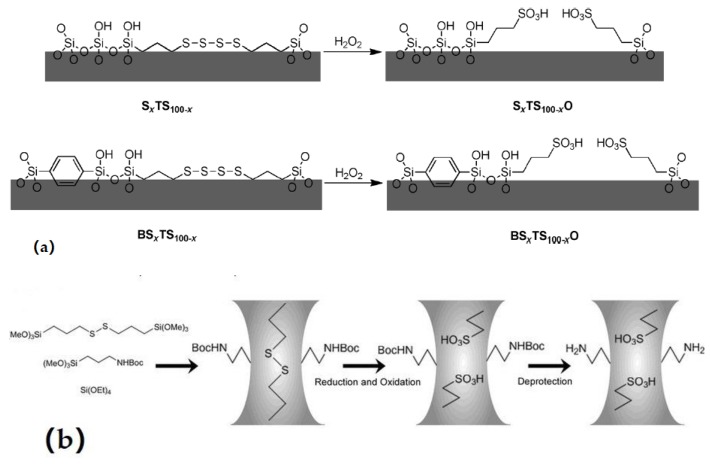
Examples of polysulfide linkers used in sulfonic acid PMOs. (**a**) Sulfonic acid functionalization of PMOs via oxidation of tetrasulfide bridges. Reproduced with permission from WILEY-VCH Verlag GmbH & Co. KGaA, Weinheim, from Reference [106]; copyright (2013). (**b**) Synthesis of bifunctional PMO via co-condensation of a bridging disulfide group and a protected aminopropyl group. Cleavage of the disulfide bridging group with sodium borohydride, followed by oxidation with hydrogen peroxide, yields sulfonic acid groups. Removal of the protecting group generates basic and acidic functionalities in a single material. Reproduced with permission from WILEY-VCH Verlag GmbH & Co. KGaA, Weinheim, from Reference [109]; copyright (2010). Note that bond geometries are not chemically accurate.

The use of disulfide [105] or tetrasulfide [106] bridging silanes (Scheme 7a,b), which open upon oxidation with HNO_3_ or H_2_O_2_, respectively, to form sulfonic acids, is an alternative attractive route to introduce acid groups within the walls of PMO materials. H_2_O_2_ is reported to be a better oxidizing reagent than HNO_3_ for the oxidation of framework disulfides [107], resulting in less degradation of the final material. The stability of sulfonic acid PMO materials produced from bridged tetrasulfides was found to decrease with increased sulfide loading, with loss of meso structure observed [106,108]. The use of disulfide bridging silanes does offer an interesting strategy to develop bifunctional catalysts with partitioned acid and base sites, via co-condensation of bridged disulfides with aminopropyl silanes (Scheme 7b) [109]; methods to prepare spatially orthogonal materials are of growing interest for cascade reactions [110]. Post modification of the organic bridge itself offers another route to introduce functionality, for example, a reaction with H_2_SO_4_ proved successful at sulfonating the phenylene or biphenylene bridge in PMO materials [46], but requires quite aggressive conditions (50% SO_3_/H_2_SO_4_ at 105–110 °C).

**Scheme 8:**
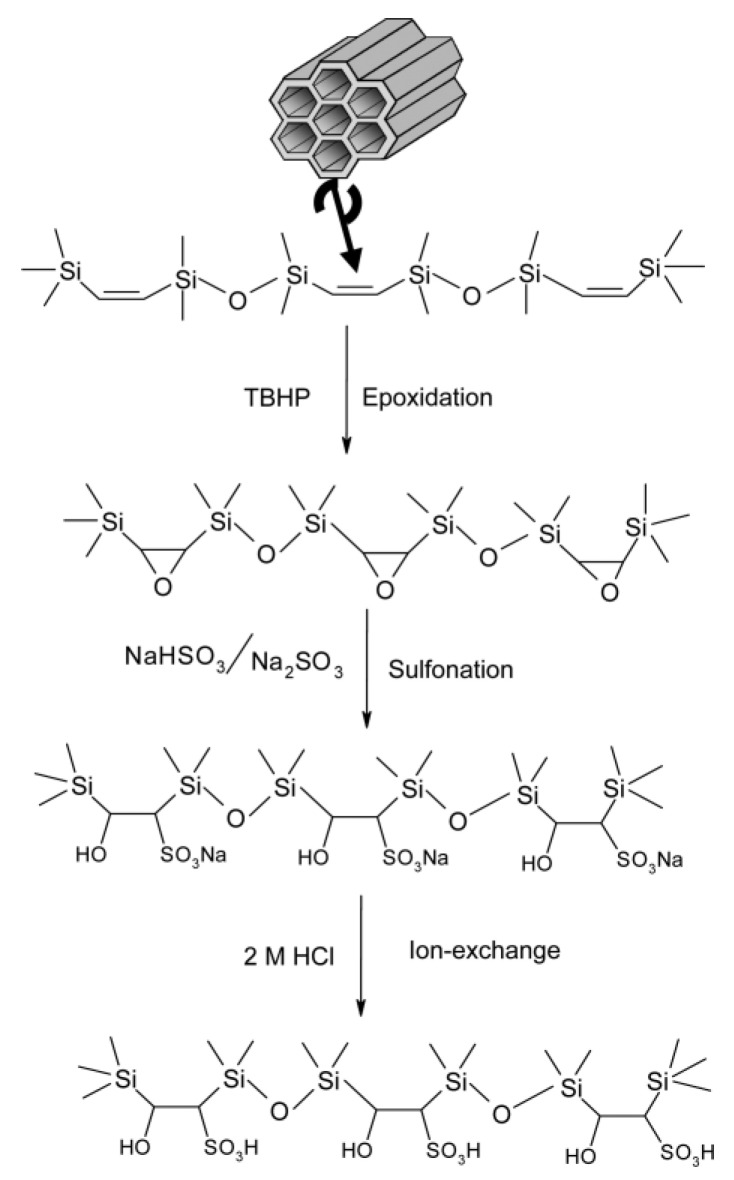
Schematic representation of sulfonic acid functionalization of Hexagonal mesoporous ethenylene-silicas (HMEs). Adapted from Reference [113] with permission; copyright (2013) American Chemical Society.

Another more controlled approach to incorporate sulfonic acid functionality involves the use of ethylene-bridged PMO frameworks, which can undergo alkylation, bromination, or Diels–Alder reactions, which facilitate sites for subsequent derivatization. Arene sulfonic acid groups were introduced into ethylene framework PMOs via Diels–Alder reaction of the ethylene group with benzocyclobutene to form a phenylene species [111], or alkylation with phenyl using homogeneous AlCl_3_ [112]; subsequent sulfonation with H_2_SO_4_ successfully produced sulfonic acid groups. Alternatively, ethylene bridges in PMO materials can undergo epoxidation followed by sulfonation using bisulphite/HCl to form sulfonic acid [113] (Scheme 8), or, if subjected to bromination, can initiate a Grignard reaction with 3-chloro-1-propanethiol, followed by oxidation to form tethered propyl sulfonic acid groups [114]. Another approach to introduce sulfonic acid groups is via grafting of 2-(3,4-epoxycyclohexyl)ethyltrimethoxysilane or 3-glycidoxypropyltrimethoxysilane, whose epoxy groups react with sodium sulfite under mildly oxidizing conditions to form sulfonic acids [115].

Grafting of 1,2,2-trifluoro-2-hydroxy-1-trifluoromethylethane sulfonic acid β-sultone was reported for the production of perfluoroalkyl sulfonic-acid-derivatized ethyl-bridged PMO materials [92]. While incorporation of perfluoroalkylsulfonic acid groups should lead to materials with stronger acidity and enhanced hydrophobic properties, stability of the Si–O–C group and associated leaching is a concern [116].

**Figure 3:**
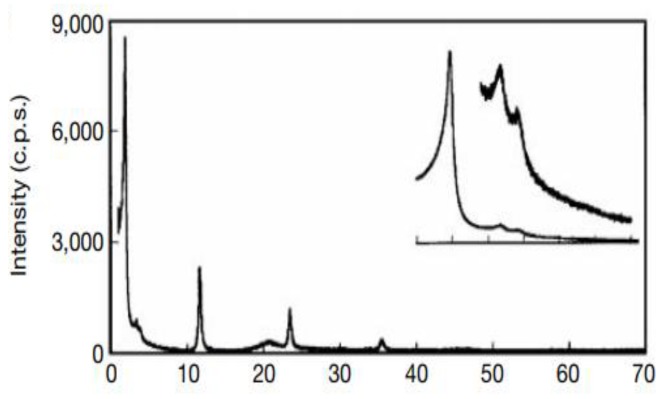
X-ray diffraction (XRD) patterns of PMO with crystal-like walls. Reprinted with permission from Springer Nature Customer Service Centre GmbH: Springer Nature [118]; copyright (2002).

Sulfonic acid PMOs were also synthesized with highly crystalline walls, originating from the periodic structural arrangement of organic groups in the pore walls [115], which tends to be observed when phenylene-bridged materials are employed [117]. Crystalline pore walls give PMOs enhanced hydrothermal stability when compared to PMOs with disordered walls. Inagaki et al. first demonstrated the formation of crystal-like pore walls (Figure 3) in phenyl-bridged PMO [118]. Yang and co-workers employed co-condensation routes to synthesize sulfonic-acid-functionalized phenyl-bridged mesoporous PMO materials with crystalline walls which exhibit strong hydrophobicity and excellent activity for esterification reactions [78]. Sulfonic-acid-derivatized PMOs are reported to be resistant toward deactivation in aqueous media compared to amorphous counterparts for the acid-catalyzed condensation of indole with benzaldehyde, and glycerol etherification [119,120]. ^1^H-NMR studies demonstrated that the activity of sulfonic acid materials is related to acid strength, which is lowered by the presence of water [120], and suggested that the phenyl rings in PMO materials inhibit solvation of framework acidic sites in water.

### 3.2. Application in Acid-Catalyzed Reactions

Sulfonic-acid-derivatized PMOs attracted significant interest as solid Brønsted acid catalysts for a variety of organic transformations including fructose dehydration to 5-HMF [121], alcohol acetylation [114,122], condensation reactions of indole with benzaldehyde [123], organic acid esterification [59,78,102,113,124], triglyceride transesterification reactions [54,103], and the protection/deprotection of alcohols with tetrahydrofuran [39].

The impact of the type of organic bridging groups, as well as the method of introducing sulfonic acid groups via MPTMS precursors in PMOs, was explored for the liquid-phase condensation of phenol with acetone to form bisphenol A (Table 1, entry 1). Materials with grafted sulfonic acid groups were found to be more active than those introduced via co-condensation, which was attributed to improved accessibility of acid sites in the former owing to preferential grafting near pore openings [125]. For both grafted and co-condensed sulfonic acids, ethyl-bridged materials show higher catalytic activity than phenyl-bridged materials, which was attributed to favorable hydrophobic properties of the material, coupled with their higher surface and improved accessibility of acid sites [43]. Varying the amount of ethyl bridges in PMOs (while keeping the sulfonic acid loading constant) has a significant impact on sulfonic acid activity for butanol dehydration to dibutyl ether. The maximum dibutyl ether yield was observed for sulfonic acid materials prepared from PMO materials having a molar ratio of 1,2-*bis*-(trimethoxysilyl)ethane/(tetramethoxysilane + 1,2-*bis*-(trimethoxysilyl)ethane) of 25 in the initial gel, which gave materials with optimal hydrophobicity to eliminate water from the acid site, and highest acid-site loadings and surface areas [126].

The advantage of hydrophobic organosilica frameworks over SBA-15 was also observed in the acetalization of heptanal by 1-butanol, where water is formed as byproduct [122]. The acidity and hydrophobicity of phenyl-bridged Ph-PMO–PrSO_3_H was explored for acid-catalyzed condensation of indole with benzaldehyde, with activity found to correlate with support hydrophobicity and also a co-operative effect between adjacent sulfonic acid groups [123]. The effect of acid density in sulfonic-acid-derivatized organosilica nanotubes was also explored for cellobiose hydrolysis to glucose (Table 1, Entry 2), with 90% conversion and 95% selectivity to glucose reported [127]. Propyl and arene sulfonic-acid-functionalized ethyl-bridged PMOs [63], and propylsulfonic acid phenylene-bridged PMO with crystal-like pore walls [117] were also been employed for fructose dehydration to HMF in dimethyl sulfoxide (DMSO) and water, respectively (Table 1, Entry 3), with superior dehydration rates observed over PMO materials compared to conventional mesoporous sulfonic acid silicas in all cases. The influence of morphology of sulfonic acid PMO materials was also explored for the esterification of levulinic acid and furfuryl alcohol (Table 1, Entry 4). By varying the synthesis methodology, PMO with spherical, hollow tubular morphology was prepared, and found to exhibit superior activity in comparison to the periodic hexagonal mesoporous counterpart [128].

**Scheme 9:**
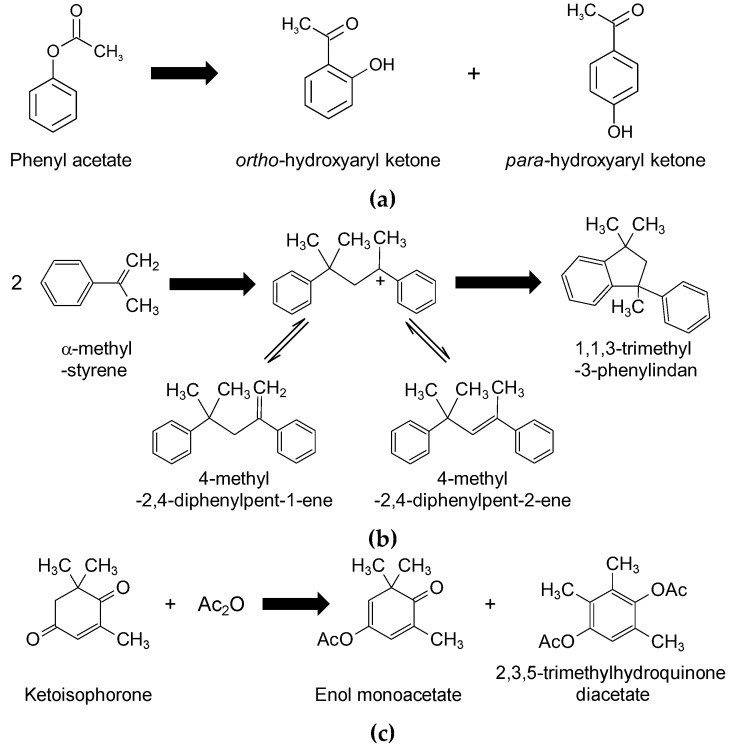
Reaction schemes for (**a**) Fries rearrangement of phenyl acetate, (**b**) dimerization of α-methylstyrene, and (**c**) rearrangement–aromatization of ketoisophorone.

The catalytic performance of sulfonic acid PMOs prepared from modification of bridging groups in PMOs was also reported. Materials prepared from sulfonation of phenyl groups introduced via a Diels–Alder reaction with ethylene-bridged PMOs were active for several acid-catalyzed reactions spanning esterification, pinacol–pinacolone rearrangement, and Beckmann rearrangements [111]. The application of sulfonic acids prepared from Grignard reaction of ethylene-bridged PMO materials exhibit excellent activity for the esterification of glycerol (Table 1, Entry 5), with comparable performance to commercial acid resins, but additional stability upon recycling [114]. The catalytic performance of sulfonic acids prepared via sulfonation of phenylene-bridged PMOs using chlorosulfonic acid [PMO(benzene)–SO_3_H] was compared with PMO materials prepared from co-condensation of phenylene and MPTMS and H_2_O_2_ oxidation [PMO(benzene)–PrSO_3_H] [129]. All materials outperformed conventional sulfonic acid SBA-15 or MCM-41 materials in gas-phase phenol alkylation with isopropyl alcohol and liquid-phase Fries rearrangement (Scheme 9a). [PMO(benzene) –SO_3_H] also exhibited higher activity and selectivity than [PMO(benzene)–PrSO_3_H] toward formation of the cyclic indan derivative during the dimerization of α-methylstyrene (Scheme 9b), owing to stronger acidity of arene sulfonic acid groups. Likewise, in the rearrangement–aromatization of ketoisophorone (Scheme 9c), [PMO(benzene)–SO_3_H] produces higher yields of more challenging 2,3,5-trimethylhydroquinone diacetate product, as compared to [PMO(benzene)–PrSO_3_H], which favors formation of more facile enol monoacetate [129]. The superiority of sulfonic acid PMOs compared to conventional mesoporous sulfonic acid silicas was also demonstrated for Claisen–Schmidt condensation of acetophenone with benzaldehyde, where the hydrophobic characteristics of the pore walls also improves catalyst performance [130].

**Scheme 10:**
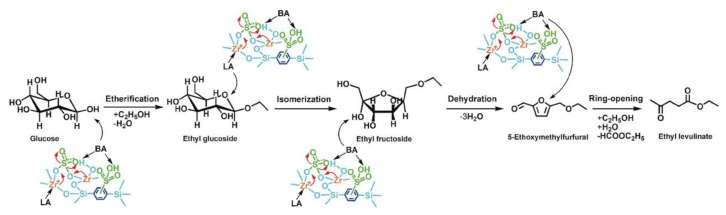
Reaction pathway of the SO_4_^2−^/ZrO_2_-PMO–SO_3_H-catalyzed conversion of glucose to ethyl levulinate in ethanol medium. Reproduced with permission from WILEY-VCH Verlag GmbH & Co. KGaA, Weinheim, from Reference [133]; copyright (2018).

Sulfonic acids were also grafted along with other functionalities and used for chemical transformations, with propylsulfonic acid-anchored isocyanurate bridging periodic mesoporous organosilica synthesized for the one-pot synthesis of *bis*-(indolyl)methane [131]. In addition, sulfonic-acid-grafted PMO materials were used in conjunction with metal or metal complexes, such as gold(I)-*N*-heterocyclic carbene, which was successfully immobilized and explored for hydration of diphenylacetylene [132]. A sulfated zirconia-doped sulfonic acid PMO material was also reported, which offered tunable Brønsted and Lewis acidity and was explored for the cascade synthesis of ethyl levulinate synthesis from glucose and ethanol (Scheme 10). The cascade proceeds via glucose-ethyl glucoside-ethyl fructoside-5-ethoxymethylfurfural-ethyl levulinate, in which Bronsted acidity is required for the etherification and dehydration steps, while Lewis acidity promotes isomerization pathways. Hydrophobic PMO-based catalysts were found to be less susceptible to deactivation owing to inhibition of water byproduct adsorption [133].

In summary, sulfonic-acid-derivatized PMO materials were explored in a wide range of acid-catalyzed reactions and show promise for improving the water tolerance compared to sulfonic acid silicas. An accurate comparison of the diverse range of materials reported to date is, however, hampered by changes to the porosity of materials with different synthetic approaches. The lack of information regarding changes in acid strength or quantification of hydrophobic properties in many studies is an area where further research is required.

### 3.3. Application of Sulfonic Acid PMOs in Biofuel Synthesis

**Scheme 11:**
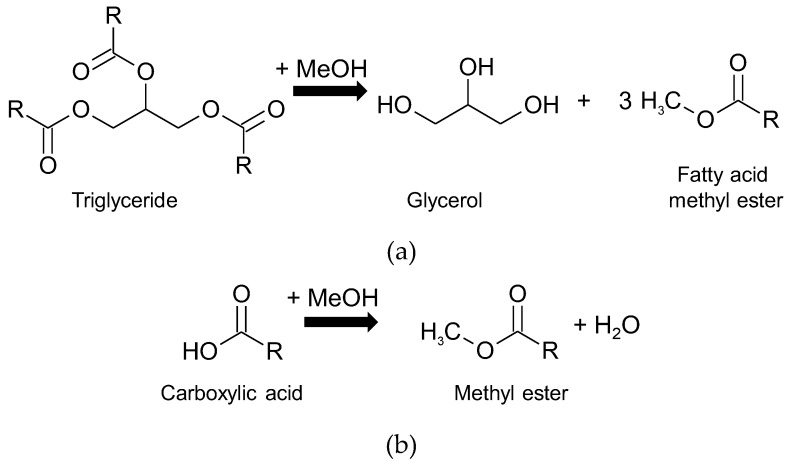
Reaction schemes for (**a**) triglyceride transesterification to fatty-acid methyl esters (R = C_11_–C_17_ saturated or unsaturated hydrocarbon chains) and (**b**) carboxylic acid esterification to methyl esters, where R = C_1_–C_2_ for bio-oil pretreatments, or C_11_–C_17_ saturated or unsaturated hydrocarbon chains for biodiesel synthesis.

The development of new solid acids for biofuel synthesis is a topic of great interest, which requires careful tuning of catalyst structure and composition to aid accessibility and strength of active sites for transesterification of bulky triglycerides [134], and esterification of fatty acids [135,136] in the context of biodiesel production, or esterification of short-chain acids for bio-oil pre-treatments during upgrading [137] (Scheme 11).

Hydrothermal stability and hydrophobicity are key design parameters to be considered when solid acids are used for biomass conversion and bio-oil upgrading, where appreciable water content in feedstocks is unavoidable. Strongly bound water can weaken acid-site strength leading to catalyst deactivation or promoting undesired hydrolysis side reactions, limiting product yields in condensation or esterification reactions. While mixed oxides [138], hydrotalcites [139], and mesporous sulphated zirconia [140] are promising materials, sulfonic-acid-derivatized mesoporous silicas offer significant versatility for esterification [54,134,136], enabling control of hydrophobic properties via co-derivatization with inert alkyl or phenyl groups [33,141].

Mesoporous silicas are generally hydrophilic materials, owing to the presence of a large quantity of surface silanol groups, which favors strong adsorption of polar molecules [142], and competitive adsorption between water of polar solvents can reduce catalytic activity [143]. Incorporation of hydrophobic groups via grafting can reduce the sites available for sulfonic acid attachment, and, as a consequence, acid loadings can be limited [144]. Hydrophobicity in PMOs originates from the bridging groups in the pore walls; therefore, they do not compete with anchoring sites for active sites. PMOs are, thus, attractive materials to generate hydrophobic sulfonic acid catalysts for sustainable biofuel production, with reactions spanning dehydration, esterification, transesterification, and condensation explored.

PMO-based catalysts attracted significant attention for application in esterification and transesterification reactions for the conversion of fatty acids and triglycerides in non-edible oils to biodiesel [145]. Esterification is an important pre-treatment for reducing the acidity of oleaginous feedstocks through carboxylic acid removal and is also important in the context of upgrading of fast pyrolysis-derived bio-oils which contain high levels of C_1_–C_3_ organic acids [140]. The corrosive nature of these bio-oils is detrimental for the lifetime of deoxygenation catalysts used in their downstream reforming to transportation fuels; hence, pre-treatment processes are required to neutralize their acidity, thereby improving oil stability [137,146]. Generation of water as a byproduct during esterification can be problematic owing to the reversibility of the reaction, and ease of ester hydrolysis upon water accumulation around the acid site. Efficient hydrophobic solid catalysts are, thus, in high demand for esterification reactions, wherein the incorporation of organic groups within mesoporous silica frameworks of a PMO weakens water adsorption near the catalytically active site.

The impact of increased hydrophobic characteristics was studied by Liu et al., who confirmed that sulfonic-acid-derivatized ethyl-bridged PMOs are stable in water and exhibit enhanced activity for the esterification of acetic acid with ethanol [147]. Sulfonic-acid-functionalized phenyl-bridged PMO with crystalline pore walls was reported to outperform commercially available Nafion resins in the esterification of acetic acid with ethanol. Enhanced activity is attributed to a combined effect of improved acid site accessibility from crystalline mesoporous walls, and the hydrophobicity of bridging phenyl groups in PMO materials [78]. Sulfonic acid PMO materials were also prepared from tetrasulfide-bridged silane precursors, which are proposed to form sulfonic acid headgroups in close proximity, which aids co-operative interactions to enhance acidity [106]. Resulting solid acids were explored in the esterification of a range of aliphatic and aromatic organic acids with primary and secondary alcohols and benzyl alcohol. For short-chain acids/alcohols, catalyst activity was comparable to Amberlyst resins; however, PMOs significantly outperformed resins when larger more hydrophobic reactants were employed, such as the reaction of octanoic acid with secondary alcohols and the synthesis of various benzyl esters. Lopez et al. also reported the application of sulfonic acid ethyl-bridged PMOs in which the acid group was introduced using a thiol-functionalized *bis*-silane precursor. These materials exhibited excellent activity for the esterification of benzyl alcohol with acetic acid, with good reusability, and they outperformed commercial acidic resins [68].

Sulfonic acid PMO materials were also employed for the transesterification of triglycerides [54], using different types of oils to produce biodiesel [103], with their excellent water tolerance desirable to overcome problems associated with high water content present in non-edible oils and waste cooking oils. Karimi and co-workers used ethyl- and phenylene-bridged PMOs functionalized with sulfonic acid for biodiesel production from sunflower, corn, canola, and refined olive oil [103]. While ethyl-bridged PMO sulfonic acids were more active than phenylene counterparts, the latter exhibited superior water tolerance when reactions were deliberately spiked with 20 wt.% water, with the biodiesel yield decreasing by only 20% for the latter compared to 60% for the former [103]. In a subsequent study, the authors used similar catalysts for esterification of fatty acids with alcohols, and again reported that ethyl-bridged sulfonic acid PMOs exhibit superior activity relative to phenyl-bridged materials, which they attributed to differences in hydrophobic characteristics [148]. However, when these catalysts were used in the acylation of 1,3-butanediol with dodecanoic acid, a balance of hydrophobicity/hydrophilicity was claimed to be necessary to direct selectivity toward mono- and diacetylated products [148]. Sulfonic-acid-containing ionic liquid (IL)-based PMOs were also used for carboxylic acid esterification with alcohols, in which the motivation was to explore whether co-operative interactions could be induced between sulfonic acid and tethered alkylimidazolium to promoted esterification activity [149]. While these materials exhibited superior activity to conventional sulfonic acid SBA-15, attributed to their enhanced hydrophobicity, no comparison with a sulfonic-acid-derivatized ethyl-bridged PMO was made to assess the impact of the IL component. While sulfonic acid PMOs synthesized from the sulfonation of 2-(3,4-epoxycyclohexyl)-ethyltrimethoxysilane precursors show excellent catalytic activity for esterification, acylation, and condensation reactions, compared to conventional sulfonic acid MCM-41, no comparison with conventional sulfonic acid PMOs using an MPTMS precursor was made to assess the benefit of using alternative methods to introduce sulfonic acid groups [115].

While these studies demonstrate a benefit of using PMO-based materials as supports for sulfonic acid catalysts, few studies attempted to quantify the magnitude of changes in hydrophobic characteristics with the amount or type of framework bridging group. The hydrophobic properties of porous materials are often empirically defined, hampering abilities to tailor catalyst surfaces in an informed manner. Simple contact-angle measurements are ill-suited to the analysis of powdered solids, since particle morphology/porosity can influence the shape of probe droplets and their absorption. To address this, Pirez et al. studied the esterification activity of sulfonic-acid-grafted PMOs prepared with 1,2-*bis*-(triethoxysilyl)ethane (BTSE) or 1,4-*bis*-(triethoxysilyl)benzene (BTSB) bridging silanes, in which the impact of varying the bridging silane content from 25–50 mol.% (named E25, E50, and B25, B50, respectively) was explored. Resulting surface adsorption characteristics and hydrophobicity was quantified by inverse gas chromatography (IGC) and correlated with catalyst activity in fatty-acid esterification [59].

Inverse gas chromatography (IGC) was employed to quantify changes in hydrophobicity, as it is a powerful technique to probe the non-polar and polar surface interactions of materials with adsorbates at a molecular level, enabling calculation of thermodynamic properties including surface energy, acid/base characteristics, free energy of adsorption, and heat of adsorption. The surface energy of an adsorbent ($\gamma_{}^{S}$) is given by the sum of dispersive ($\gamma_{D}^{S}$) and specific free energy ($\gamma_{SP}^{S}$) components as shown in Equation (1).

(1) $$\gamma_{}^{S}=\;\gamma_{D}^{S}+\gamma_{SP}^{S}.$$

Calculation of the standard free energy of adsorption $-\Delta G_{ads}^{}$ is also possible using the dispersive and specific energies for adsorption of probe molecules according to Equations (2) and (3) [150], wherein $V_{N}^{}$ is the specific retention volume of the adsorbate.

(2) $$\left[-\Delta G_{ads}^{}\right]=\;[-\Delta G_{ads}^{D}]+[-\Delta G_{ads}^{SP}]$$

(3) $$\Delta G_{ads}^{}=\;-RT.\ln V_{N}^{}+\;constant$$

The adsorption of non-polar molecules occurs via non-specific interactions, or London attractive forces with the surface, whereas, for polar molecules, adsorption also involves specific contributions originating from, e.g., acid/base, hydrogen, or π-bonding interactions. Adsorption of a homologous series of, e.g., C_6_ to C_10_ alkane probe molecules allows determination of dispersive surface energies (${}_{D})$ from non-specific van der Waals interactions. The dispersive components of surface energies for the adsorbent and probe molecules ($\gamma_{D}^{S}$ and $\gamma_{D}^{L}$ respectively) and $RT.\ln\left(V_{N}^{alkane}\right)$ obey a linear relationship according to Equation (4) [151], where *N* is Avogadro’s number, *a* is the surface area of the probe molecule, and $V_{N}^{alkane}$ is the specific retention volume of an alkane with *N* carbon atoms, which is proportional to its specific retention time as measured by IGC.

(4) $$RT.\ln\left(V_{N}^{alkane}\right)=2N.a.{\left(\gamma_{D}^{S}\right)}^{1/2}.{\left(\gamma_{D}^{L}\right)}^{1/2}+constant$$

**Figure 4:**
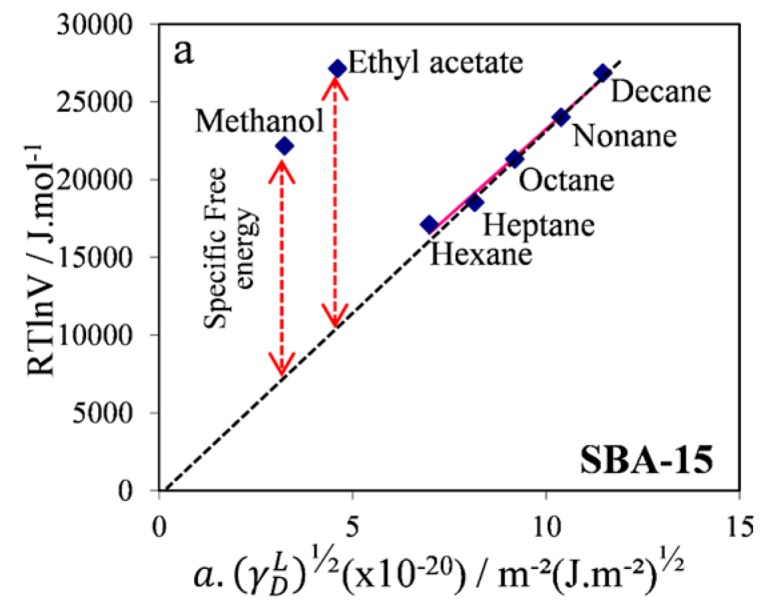
Application of inverse gas chromatography (IGC) to determining $\gamma_{D}^{S}$ and $-\Delta G_{ads}^{SP}$ for SBA-15. Adapted from Reference [59]; copyright (2014) with permission from Elsevier.

Figure 4 shows how a plot of $RT.\ln\left(V_{N}^{alkane}\right)$ against $a.{\left(\gamma_{D}^{L}\right)}^{1/2}$ for SBA-15 yields a straight line with slope $2N.a.{\left(\gamma_{D}^{S}\right)}^{1/2}$, enabling the non-specific dispersive component of the surface energy ($\gamma_{D}^{S}$) to be determined. Application of this methodology to a family of PrSO_3_H–PMO adsorbents with BTSE and BTSB bridges [152] enabled the impact of changes in organic content on $\gamma_{D}^{S}$ to be correlated. Adopting the same approach for polar probe molecules allows *RT.*ln*V* to be calculated from their measured retention time (volume), which is proportional to $-\Delta G_{ads}$ (the total free energy of adsorption). The specific free energy of adsorption $\left(-\Delta G_{ads}^{SP}\right)$ of the polar molecule is the difference between $-\Delta G_{ads}$ and the vertical intercept with the extrapolated straight line of the non-specific dispersive energies, as show in Figure 4.

**Figure 5:**
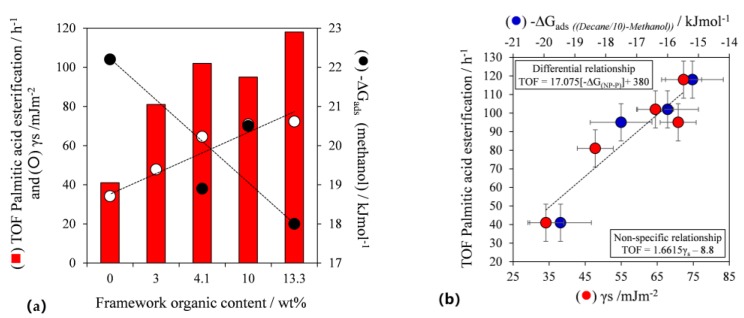
(**a**) Impact of framework organic content on PMO surface properties as determined from IGC and TOF for palmitic acid esterification with methanol. (**b**) Correlation of dispersive energy and $\left[-\Delta G_{NP-P}^{C}\right]$ for decane and methanol, with TOF for palmitic acid esterification with methanol. Adapted from Reference [59]; copyright (2014) with permission from Elsevier.

**Figure 6:**
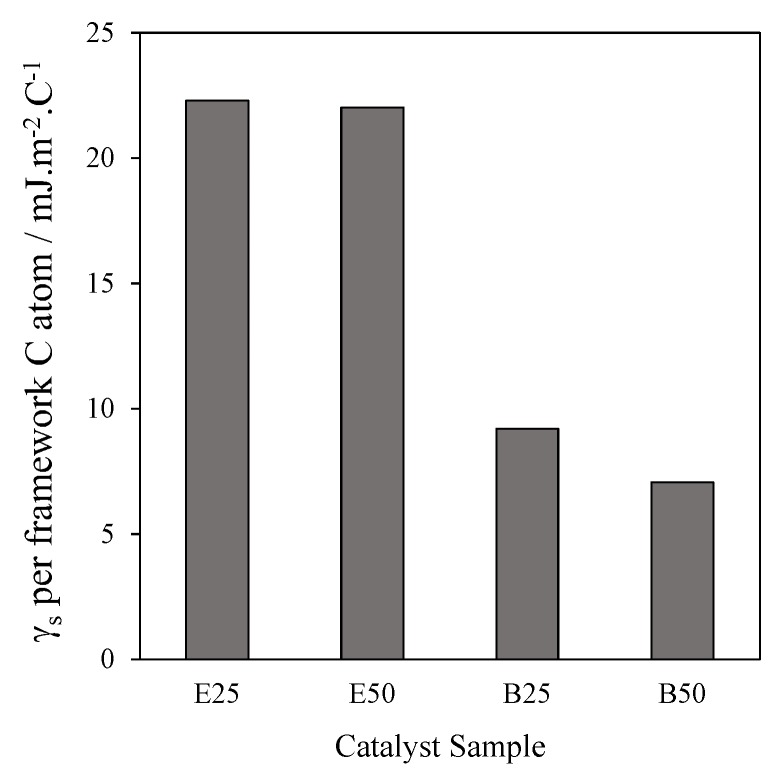
CH_2_ and CH normalized dispersive surface energies for ethyl (E25 and E50) and phenyl (B25 and B50) substituted PMO–SBA materials showing ethyl groups have a stronger influence on dispersive surface energy per CH_2_ unit than the corresponding CH sites in aromatic framework substituents. Adapted from Reference [59]; copyright (2012) with permission from Elsevier.

Esterification activity of hexanoic and palmitic acid with methanol was found to increase with framework organic content, irrespective of whether framework ethyl or phenyl groups were employed in the sulfonic acid PMO (Figure 5a). IGC analysis revealed that addition of ethyl and benzyl chains in the mesoporous silica framework increased the dispersive (non-specific) surface energy while decreasing the free energy of methanol (reactant) adsorption (Figure 5a), reflecting increased surface hydrophobicity. The linear correlation between esterification activity and non-specific energy, thus, reflects a correlation between hydrophobicity and catalytic activity. Closer examination in the relative changes in turnover frequency (Figure 5a) showed some non-linearity in activity and γ_s_ between 4.1 to 10 wt.% organic content, which corresponds to samples synthesized from 50 mol.% BTSE versus 25 mol.% BTSB, respectively. This non-linearity was suggested to originate from ethyl functions possibly being more uniformly distributed in the pore walls than bulkier phenyl groups from BTSB. In addition, differences in the electronic properties of aliphatic CH_2_ groups in BTSE will differ from the aromatic CH functions in BTSB, resulting in further perturbation of surface adsorption properties. Indeed, when the change in γ_s_ was normalized to the number of C atoms introduced from BTSE or BTSB (Figure 6), values for CH­_2_ in E50 and E25 were comparable, but three times higher than for CH in B50 and B25. Thus, ethyl groups appear to be more effective than phenyl moieties in modifying the surface energy, which is in accord with other work that suggests ethyl groups are superior at imparting hydrophobic properties [148].

IGC is, thus, a powerful tool to deduce the surface adsorption properties of organically functionalized materials such as PMO, and was also extended to materials used in esterification pre-treatments of pyrolysis bio-oil [32]. Here, IGC demonstrated the relationship between catalyst surface polarity (*X*_p_), calculated as the ratio of the polar surface energy to the total surface energy (Equation (5)), with hydrophobicity and water tolerance in esterification reactions.

*X*_p_ = γ_S_^SP^/(γ_S_^SP^ + γ_S_^D^). (5)

Surface polarity decreased with increased carbon content in hybrid organic–inorganic sulfonic acid silicas, and correlated with increased activity for esterification of acetic acid, suggesting surface polarity is another useful parameter for future quantification of surface hydrophobicity in PMO materials [32].

## 4. Concluding Remarks

Since the successful synthesis of PMO materials was first reported [40], significant advances were made in their catalytic applications, where their desirable hydrophobic characteristics and hydrothermal stability proved beneficial. Subsequent innovative design of bridging silanes enabled functional groups to be introduced in a controlled manner, wherein the catalytically active sites are incorporated into the bridging organic silane. Alternatively, co-condensation of functional silanes during synthesis of the PMO, or post modification of PMO materials was successfully employed to develop derivatized materials for catalytic applications.

The ability to control hydrophobic properties in catalytic materials is of special importance for the development of next-generation catalysts for biorefinery applications, spanning platform chemical production, biodiesel synthesis from oleaginous feedstocks, and upgrading of bio-oils derived from fast pyrolysis or hydrothermal liquefaction. Sulfonic-acid-derivatized PMO materials were successfully explored in a range of acid-catalyzed reactions spanning dehydration, acetylation, condensation, esterification, and transesterification reactions, with significant enhancements in performance observed when benchmarked against conventional mesoporous sulfonic acid catalysts or commercial resins. Use of designer tetrasulfide bridging silanes to introduce sulfonic acid groups was also suggested to promote co-operative effects in catalytic reactions, owing to optimal spatial separation of acid centers.

While several classes of sulfonic acid PMO materials were described based upon different framework organic groups (e.g., bridging ethyl, ethenyl, or phenyl groups), functionalized bridging silanes (e.g., di- or tetrasulfide), or alternative modification methods using organo-thiol groups (e.g., use of mercaptopropyltrimethoxysilane, derivatization of epoxides, or direct sulfonation of aromatic groups), few studies benchmarked results across these families to assess which routes are superior. More detailed investigations to quantify the effect of hydrophobicity and impact of surface polarity on competitive adsorption of reactants in bimolecular reactions are required to guide future formulations. IGC demonstrates promise as a powerful technique for assessing non-specific and specific surface energies of PMO materials, and surface polarities of organic–inorganic hybrid materials, and should prove invaluable in the future for quantifying surface hydrophobicity and its impact on catalyst performance in aqueous environments and for biomass conversion.

## Figures and Tables

**Table 1:** Reaction schemes and performance of periodic mesoporous organosilica (PMO) sulfonic acids. Entry 1—condensation of phenol with acetone to bisphenol A (BPA); Entry 2—hydrolysis of cellobiose to glucose; Entry 3—dehydration of fructose to 5-HMF; Entry 4—alkyl levulinate production via esterification of levulinic acid or furfural; Entry 5—glycerol esterification with acetic acid.

Reaction Scheme	Performance	Refs
Entry 1 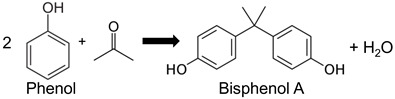	TOF ^a^ BPA production Grafted PrSO_3_H–PMO ^b^: Ethyl bridge = 17.2 h^−1^ Phenyl bridge = 7.6 h^−1^ PrSO_3_H–SBA = 8.8 h^−1^	[43]
Entry 2 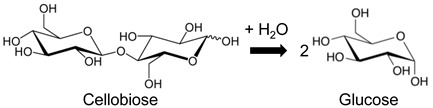	TOF cellobiose conversion PrSO_3_H PMO nanotubes: Ethyl bridge = 7.5–10.4 h^−1^ Phenyl bridge = 7–12.1 h^−1^ PrSO_3_H/SiO_2_ NT ^c^ = 7.8 h^−1^	[127]
Entry 3 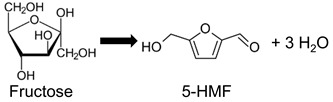	TOF HMF production ^b^ Ethyl-bridged PMO phenyl-SO_3_H = 103 h^−1^ ^b^ SBA-phenyl-SO_3_H = 41 h^−1^ TOF fructose conversion ^c^ Ethyl-bridged PMO–PrSO_3_H= 520–910 h^−1^ ^c^ Phenyl-bridged PMO–PrSO_3_H = 350 h^−1^	[63] ^d^ [117] ^e^
Entry 4 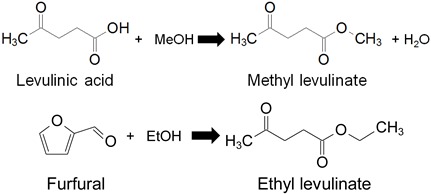	TOF levulinic acid conversion Ethyl-bridged PMO phenyl-SO_3_H = 109 h^−1^ Amberlyst-15 = 18 h^−1^ TOF furfural conversion Ethyl-bridged PMO phenyl-SO_3_H = 10.5 h^−1^ Amberlyst-15 = 4 h^−1^	[128]
Entry 5 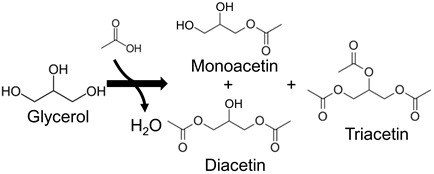	Conversion/Selectivity Acetylation yield = 80% Monoacetin selectivity = 94% Diacetin selectivity = 6%	[114]

^a^ TOF=Turnover frequency; ^b^ PrSO_3_H=propyl sulfonic acid; ^c^ NT=nanotube; ^d^ productivity estimated from S content in dimethyl sulfoxide (DMSO) at 120 °C; ^e^ water with Methyl isobutyl ketone (MIBK)/2-butanol (70/30) at 160 °C.
